# HTK Is a Viable UW Alternative for Hypothermic Oxygenated Machine Perfusion of Liver Grafts Supporting a Single-Solution Protocol

**DOI:** 10.3390/jcm15010112

**Published:** 2025-12-24

**Authors:** Jule Dingfelder, David Pereyra, Moriz Riha, Nikolaus Becker, Laurin Rauter, Hubert Hackl, Julian Flavio Müller, Felix Hammer-Purgstall-Bernd, Monika Aiad, Jakob Eichelter, Patrick Starlinger, Gerd R. Silberhumer, Andreas Salat, Gabriela A. Berlakovich, Georg Györi, Thomas Soliman

**Affiliations:** 1 Department of General Surgery, Division of Transplantation, Medical University of Vienna, 1090 Vienna, Austriadavid.pereyra@meduniwien.ac.at (D.P.); nikolaus.becker@meduniwien.ac.at (N.B.); gabriela.berlakovich@meduniwien.ac.at (G.A.B.);; 2 Department of General Surgery, Division of Visceral Surgery, Medical University of Vienna, 1090 Vienna, Austria; 3 Department of Surgery, Division of Hepatobiliary and Pancreas Surgery, Mayo Clinic, Rochester, MN 55905, USA; 4 Institute of Bioinformatics, Biocenter, Medical University of Innsbruck, 6020 Innsbruck, Austria; hubert.hackl@i-med.ac.at; 5 Centre of Physiology and Pharmacology, Medical University of Vienna, 1090 Vienna, Austria

**Keywords:** machine perfusion, HOPE, perfusion solution, single-solution strategy

## Abstract

**Background and Aims:** Hypothermic oxygenated machine perfusion (HOPE) improves outcomes in orthotopic liver transplantation (OLT), but reliance on University of Wisconsin machine perfusion solution (UW-MPS) increases costs and logistical burden. Histidine-tryptophan-ketoglutarate (HTK) has potential as a single-solution alternative for HOPE. This study evaluated the safety and efficacy of HTK versus UW-MPS during HOPE. **Methods:** A retrospective, propensity score-matched cohort study including 46 patients who received donation after brain death (DBD) grafts that were preserved with HOPE at the Medical University of Vienna between May 2018 and October 2024 was conducted. A total of 23 patients received grafts perfused with HTK; another 23 patients transplanted with organs perfused with UW-MPS were matched based on recipient age and sodium model of end-stage liver disease score, donor age and sex, cold ischemia time, and perfusion time. Postoperative outcomes, perfusion parameters, and cost differences were assessed. **Results:** The HTK and UW-MPS cohorts demonstrated comparable perfusion dynamics and vascular resistance. While arterial pressure and flow were higher in the UW-MPS group, clinical outcomes—including early allograft dysfunction (47.8% each), ICU stay, and comprehensive complication index—were statistically similar. A trend toward fewer biliary complications (13.0% vs. 30.4%) and reduced hemodialysis requirement (17.4% vs. 30.4%) was observed in the HTK group. Use of HTK reduced perfusion-related costs by approximately EUR 560 per procedure. **Conclusion:** HTK is a viable alternative to UW-MPS during HOPE in OLT of DBD grafts, offering comparable short-term outcomes and relevant cost savings. Prospective studies are warranted to validate these findings and explore broader applications of single-solution perfusion strategies.

## 1. Introduction

Due to its beneficial effects on patient outcome, hypothermic oxygenated machine perfusion (HOPE) of donor livers has gained increased recognition in the field of liver transplantation (OLT) in recent years [1,2,3,4,5]. Its beneficial effect on reduction in biliary complications, perioperative morbidity, and length of hospitalization is backed up by several randomized controlled trials and long-term data [3,4,6,7]. Currently, the University of Wisconsin (UW) solution remains the most used perfusion solution for HOPE. Although modifications of the original formulation have led to an improved machine perfusion variant (UW-MPS), the original purpose of this solution was preservation of organs during static cold storage (SCS). Similarly, histidine-tryptophan-ketoglutarate (HTK) is a widely used preservation solution that is primarily used for SCS.

Of note, clinical protocols have recommended strict consistency in the utilization of preservation solutions throughout the entire transplantation process—from organ retrieval to implantation—regardless of the specific solution used in each step. This recommendation was based on the rationale that the significant differences in electrolyte composition, colloid content, buffering capacity, and metabolic substrates among the available solutions may impair their efficacy in organ preservation [8].

However, the introduction of HOPE disrupted this paradigm in routine clinical practice. With UW-MPS being the only commercially available solution specifically recommended for machine perfusion, transplant centers frequently faced situations in which donor organs that were procured using different solutions required the transition to UW-MPS for HOPE [9]. Although no harmful effects have been proven, using different preservation solutions at each stage of transplantation adds logistical challenges and increases the overall cost of using HOPE.

In response, several centers have initiated efforts to adopt a single-solution strategy [8,10]. HTK displays promising characteristics for such an approach; besides its low viscosity, which might improve pump mechanics during perfusion, its relatively low cost, and its long-standing, widespread use in clinical transplantation are attractive features [10,11].

The present investigation aimed to evaluate the feasibility and efficacy of using HTK as a single-solution approach in the context of HOPE for organ preservation.

## 2. Materials and Methods

### 2.1. Patients

This cohort study included patients who underwent OLT at the Medical University of Vienna between May 2018 and October 2024 who received grafts preserved via HOPE. The use of HTK during HOPE was implemented in May 2023 as a response to supply shortages of UW solution. All patients received full-organ transplantation. Patients undergoing multi-organ transplantation were excluded. All grafts were donated after brain death. All relevant data regarding inpatient care and postoperative follow-up were entered prospectively into the institution’s clinical database. Ethical approval for this study was granted by the ethics committee of the Medical University of Vienna (reference numbers EK#1610/2023 and EK#2209/2018), and the research adhered to the principles outlined in the Declaration of Helsinki (2013 revision). Written informed consent for machine perfusion-related studies and use of generated data was obtained from all subjects involved in the study.

### 2.2. Hypothermic Machine Perfusion

All organs were retrieved following established surgical protocols and preserved via SCS before being transferred to the Medical University of Vienna. During all procurements, the HTK solution was used for cold flush and storage. Following back-table preparation, grafts were subjected to HOPE. HOPE procedures utilized the LiverAssist^®^ perfusion system (XVIVO Perfusion, Stockholm, Sweden) in combination with Belzer UW-MPS or HTK, depending on the availability of perfusion solution. Perfusion was conducted at temperatures between 8 °C and 15 °C. During perfusion, flow rates in both the portal vein and hepatic artery were regulated via adaptation of applied pressure in 1 mmHg increments, and adequate oxygenation by integrated dual hollow-fiber membrane oxygenators was ensured using point-of-care testing. Of note, pressure, flow rates, and vascular resistance as indicated by the LiverAssist^®^ device were documented in 30-min intervals for both vessels. Target portal venous pressure was 3–5 mmHg. Target pressure for the arterial side was 30 mmHg until about October 2022 and 25 mmHg afterwards. Before implantation, another cold flush of the liver with either UW (not UW-MPS) solution or standard HTK, depending on the used perfusate, was performed to complete the preservation process. The choice of terminal flush solution depended on the utilized perfusate (either HTK or UW). Of note, between one and two liters of perfusate were used during back-table preparation of liver grafts, while two liters were never exceeded. For machine perfusion, two liters of perfusate were used, and a terminal flush was always performed with one liter. Accordingly, a total of five units of perfusate (HTK or UW-MPS) at each one-liter volume were billed per procedure.

### 2.3. Standard Institutional Procedures

Donor selection and organ implantation followed standard institutional procedures throughout the entire study period. Criteria for donor acceptance intended for transplantation after HOPE, including clinical assessment, laboratory evaluation, and organ quality parameters, were applied consistently and did not change over the years. Likewise, surgical implantation techniques and perioperative management, including immunosuppressive regimens, adhered to established protocols without modification during the inclusion period.

### 2.4. Outcome Parameters

Postoperatively, routine laboratory evaluation, including liver enzymes, was conducted daily. The duration of intensive care unit (ICU) stay and total hospitalization were documented. Any alterations from the normal postoperative course were followed up, and a comprehensive complication index (CCI) was calculated as previously described. Early allograft dysfunction (EAD) was defined according to Olthoff et al. [12]. Further, patients were followed up for the development of biliary complications within the first twelve months after OLT. Of note, magnetic resonance cholangiopancreatography (MRCP) was performed three to four months post-transplant as part of routine follow-up. Additionally, MRCP imaging was conducted on a case-by-case basis if clinical or laboratory signs of cholestasis emerged. Assessors were blinded to perfusate. Costs were calculated on the basis of in-house prices for preservation/perfusion solutions.

### 2.5. Statistical Analysis and Propensity Score Matching

Statistical analyses were performed using SPSS software version 27.0 (IBM Corp., Armonk, NY, USA). Normality distributions were assessed using the Shapiro–Wilk test and supported by visual examination of adequate plots. As most variables did not meet assumptions of normality, continuous variables were compared using the Wilcoxon rank-sum test. Categorical variables were compared using chi-squared tests or Fisher’s exact test when expected cell counts were less than 5. Univariate logistic regression analysis was used to evaluate the impact of date of inclusion and inclusion epoch (prior to or after adaptation of arterial perfusion pressure targets) on key outcome parameters.

Propensity score matching was performed using R version 3.4.1 (R Foundation for Statistical Computing, Vienna, Austria) with the MatchIt package. A logistic regression model including recipient age and sex, donor age, Na-MELD score, cold ischemia time (CIT), and perfusion time was used to estimate propensity scores. Nearest-neighbor 1:1 matching without a caliper and without replacement was applied. Covariate balance after matching was assessed using standardized mean differences.

Results are presented as median with interquartile range (IQR) or as absolute numbers and percentages for categorical variables. Statistical significance was defined as a two-sided *p*-value < 0.05. Due to the exploratory nature of the study, no adjustments for multiple testing were applied. Given the descriptive intent of the analysis, pointwise comparisons were used to illustrate temporal patterns rather than to draw longitudinal inferential conclusions. Boxplots were generated with outliers removed to improve the visualization of interquartile ranges.

## 3. Results

### 3.1. Propensity Score Matching

During the observational period, a total of 173 patients underwent OLT after HOPE. Of these, 23 patients received liver grafts that were perfused using HTK solution. As HTK solution was only used in organs procured via donation after brain death, only this type of donation was included in the following step. In order to account for potentially confounding factors propensity score matching was performed based on donor and recipient age, calculated lab Na-MELD, CIT prior to HOPE, perfusion time and recipient sex (Supplementary Figure S1). Accordingly, a cohort of 23 patients with similar characteristics and with use of UW-MPS during HOPE was identified (Love plot illustrating the absolute standardized mean differences for parameters used for propensity score matching can be found in Supplementary Figure S2). Patient characteristics are visualized and compared in Table 1.

### 3.2. Machine Perfusion and Flow Characteristics

**Figure 1:**
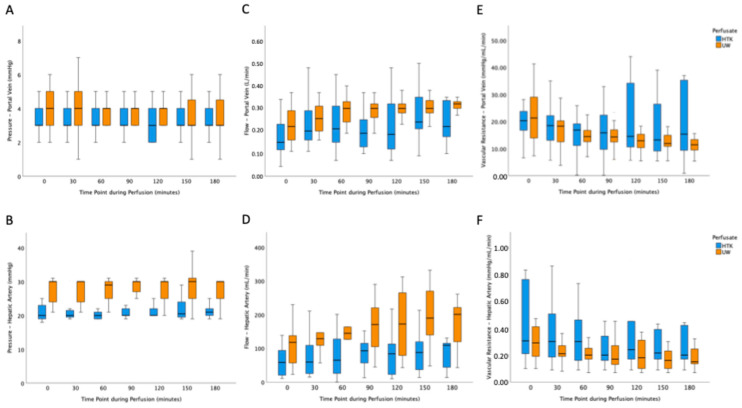
Group comparison of machine perfusion and flow characteristics. (**A**) portal vein pressure, (**B**) hepatic artery pressure, (**C**) portal vein flow, (**D**) hepatic artery flow, (**E**) portal vein vascular resistance, (**F**) hepatic artery vascular reseistence. mmHg = millimeter mercury, HTK = histidine-tryptophan-ketoglutarate, UW = University of Wisconsin solution. Outliers were excluded for visualization.

Noticeably, the majority of donor grafts were subjected to HOPE via portal vein and hepatic artery (63%) and there was no difference in distribution of single or dual HOPE between investigated cohorts. Little’s missing completely at random testing for perfusion parameters revealed that the pattern of missing values was consistent with completely at random (χ^2^ = 285.931, df = 402, *p* > 0.999). While there was no difference in portal venous pressure applied during HOPE between cohorts (Figure 1A), the applied arterial pressure and concomitantly the arterial flow rates were higher in the UW-MPS cohort at all time points (Figure 1B,D). Interestingly, portal venous flow was significantly higher in grafts perfused with UW-MPS solution between 60 and 120 min of HOPE (Figure 1C). Hepatic artery flow was higher in the UW-MPS cohort throughout perfusion (Figure 1D), which is attributable to a difference in arterial target pressure coinciding with the change in perfusate (Supplementary Table S1). The observed differences in pressure setting and observed flow rates did; however, not lead to a difference in vascular resistance of the portal vein or the hepatic artery between cohorts (Figure 1E,F). Median values are displayed in Figure 1. The pointwise comparisons reflect exploratory assessment of perfusion dynamics and were not intended as longitudinal inferential testing.

### 3.3. Postoperative Dynamics and Outcome

**Figure 2:**
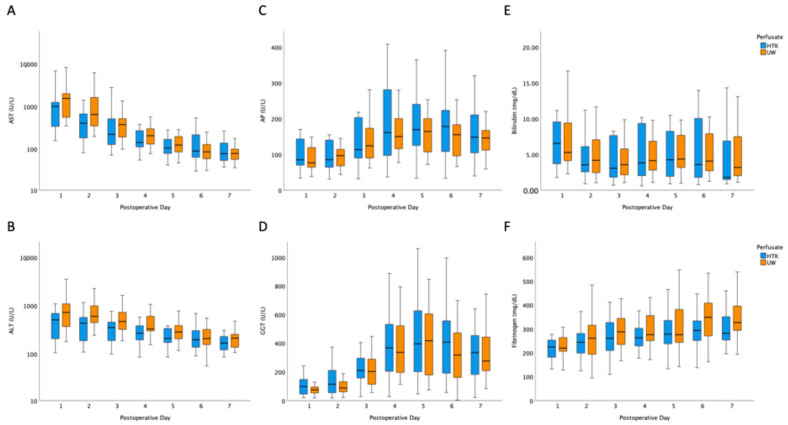
Group comparison of postoperative laboratory parameters. (**A**) AST = aspartate aminotransferase, (**B**) ALT = alanine aminotransferase, (**C**) AP = alkaline phosphatase, (**D**) GGT = gamma-glutamyl transpeptidase, (**E**) bilirubin, (**F**) fibrinogen. HTK = histidine-tryptophan-ketoglutarate, UW = University of Wisconsin solution.

First, postoperative laboratory dynamics were used for estimation of patients’ individual course. Little’s missing completely at random testing for postoperative laboratory data (χ^2^ = 187.377, df = 206, *p* = 0.820) revealed that the pattern of missing values was consistent with completely at random. With the sole exception of aspartate aminotransferase (AST) on postoperative day (POD) 3, there was no difference in transaminases (Figure 2A,B), cholestasis parameters (Figure 2C,D), bilirubin (Figure 2E), or fibrinogen (Figure 2F) between cohorts. Because the study was exploratory and not designed for formal longitudinal inference, postoperative laboratory values were compared descriptively at each time point using pointwise tests.

**Figure 3:**
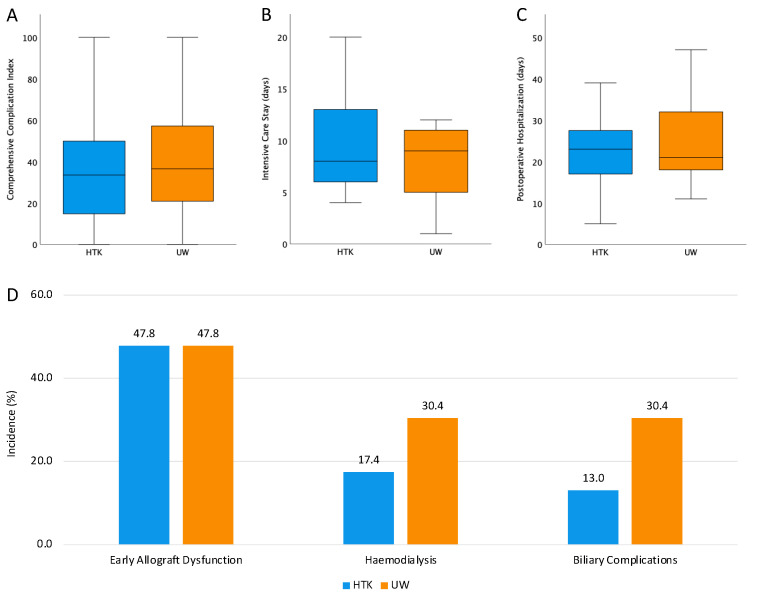
Group comparison of postoperative outcome parameters. (**A**) Comprehensive complication index, (**B**) Intensive care stay, (**C**) postoperative hospitalization, (**D**) Incidence of early allograft dysfunction, haemodialysis, and biliary complications. HTK = histidine-tryptophan-ketoglutarate, UW = University of Wisconsin solution.

In line, there was no difference in CCI (Figure 3A), duration of ICU stay (Figure 3B), and postoperative hospitalization (Figure 3C) between patients receiving grafts after HOPE with either HTK or UW-MPS. EAD was observed in 11 of 23 patients (47.8%) in both cohorts (risk difference = 0.0%, 95% confidence interval [95%CI] = −28.9–28.9%, *p* = 1.000, Figure 3D). In parallel, primary non-function was observed in one patient per cohort. Use of hemodialysis during ICU stay was more pronounced in patients receiving grafts after HOPE with UW-MPS (4 of 23 in HTK vs. 7 of 23 in UW, risk difference = −13.0%, 95%CI = −37.4–11.3%, *p* = 0.300, Figure 3D). Ultimately, biliary complications (biliary leaks and anastomotic stenoses) within the first twelve months after OLT occurred tendentially more frequently after use of UW-MPS during HOPE (3 of 23 in HTK vs. 7 of 23 in UW, risk difference = −17.4%, 95%CI = −40.7–5.9%, *p* = 0.153, Figure 3D). Routine MRCP was performed in 83% (19/23) of recipients in both groups. In the UW cohort, 17% (4/23) of patients received a symptom-triggered MRCP compared to 9% (2/23) out of the HTK cohort. No case of ischemic-type biliary lesions was observed in either group. Importantly, univariate logistic regression analysis did not reveal an association between time point of inclusion and occurrence of key outcomes (EAD: date of transplantation odds ratio [OR] = 0.988, 95%CI = 0.946–1.033, *p* = 0.596; pressure target epoch OR = 1.333, 95%CI = 0.411–4.330, *p* = 0.632; hemodialysis: date of transplantation OR = 0.959, 95%CI = 0.908–1.013, *p* = 0.131; pressure target epoch OR = 0.658, 95%CI = 0.168–2.580, *p* = 0.548; biliary complications: date of transplantation OR = 0.965, 95%CI = 0.912–1.020, *p* = 0.205; pressure target epoch OR = 0.476, 95%CI = 0.114–1.986, *p* = 0.309).

At our center, UW-MPS is purchased at a price of EUR 191, while HTK comes at a cost of EUR 79. As outlined in the methods section, a total of five units of perfusate at one liter each were billed for each case. Thereby, the use of HTK compared to UW-MPS led to a reduction in cost of EUR 560 per procedure at our center. Noticeably, this calculation only includes the type of perfusate used, as all other parameters, with cost for device and disposables being the most relevant, did not differ between cases/cohorts.

## 4. Discussion

While the overall beneficial effect of HOPE does not need further discussion, the associated costs and increased logistic complexity slow its broad implementation into routine clinical practice, especially in resource-limited settings [13,14]. A potential strategy to streamline this process is the adoption of a single-solution protocol instead of utilizing multiple preservation solutions for explantation, SCS, and end-ischemic machine perfusion. HTK is the standard cold storage solution used in Germany and Austria. Therefore, we compared HOPE using HTK versus UW-MPS as perfusion solutions; all grafts arrived cold-stored in HTK at our center. We here present the first data on the use of HTK as a single solution during organ preservation and perfusion. These findings should be interpreted in light of the exploratory design and the use of pointwise rather than longitudinal modeling approaches.

An essential requirement for a single-solution protocol is the solution’s uncompromised performance with respect to technical aspects of HOPE. Importantly, basic perfusion parameters indicating the dynamics of organ perfusion were comparable between cohorts. Venous vascular pressures required to achieve adequate graft perfusion were nearly identical between the two groups. Similarly, no statistically significant differences were observed in vascular resistance over time. Although portal venous flow showed intermittent differences at certain mid-procedural time points, these were inconsistent and likely of limited clinical relevance. The potential influence of machine readjustments during perfusion could not be definitively assessed, limiting further interpretation of these findings.

In contrast, the mean hepatic arterial pressure and, consequently, arterial flow were significantly higher in the UW-MPS group. Notably, arterial resistance remained comparable across both cohorts. Recommendations regarding arterial perfusion pressure from the manufacturer were adapted during the phase of UW-MPS use; the observed differences are therefore most likely due to variations in machine settings rather than inherent physical properties of the perfusion solutions.

The second objective of this study was to evaluate whether liver transplantation outcomes using HTK in a single-solution strategy are at least equivalent to those achieved with the conventional approach using UW-MPS. Early postoperative laboratory parameters showed no statistically significant differences between the groups. Liver enzyme levels, cholestatic markers, and synthetic liver function were nearly identical, resulting in an identical rate of 47.8% of EAD in both cohorts.

Similarly, there were no significant differences in regard to the comprehensive complication index and the duration of ICU stay. The incidence of postoperative hemodialysis was lower in the HTK group (17.4% vs. 30.4%), although this difference did not reach statistical significance. A comparable trend was observed in the rate of biliary complications within the first twelve months post-transplant (13.0% vs. 30.4%). While this difference was also not statistically significant, it is in line with a previous report that suggested a potential benefit on the development of biliary complications after SCS utilizing HTK [15]. However, other studies have reported conflicting results, underlining the need for further prospective investigations into this topic [11].

While recent evidence supports the long-term cost-effectiveness of HOPE [13], a simplified protocol could further enhance economic viability. In addition to adequate technical and clinical outcomes, our data demonstrated that the use of HTK in a single-solution approach reduces perfusion-related costs by EUR 560 per procedure, thereby contributing meaningfully to a faster amortization of HOPE-related expenditures.

The main limitation of this study lies in its retrospective design, despite propensity score matching that accounted for relevant factors potentially influencing clinical outcomes. The retrospective design is, moreover, paralleled by a sequential construction of this study, which is due to the fact that HTK was used as an alternative for UW-MPS during a moment of supply shortage. Accordingly, the current study relies on a comparison of two study cohorts that are also separated temporarily. While the time of inclusion did not seem to affect key outcome parameters, prospective investigations are relevant in order to overcome these limitations in study design. Further, the sample size was not powered for the detection of subtle to modest differences between groups (see detectable-effect illustration in Supplementary Figure S2). Some exploratory observations, such as the potential reduction in biliary complications, warrant validation through prospective, randomized controlled trials with longer follow-up. Furthermore, the design of this study was not suitable to address the safety of HTK as a perfusion solution in prolonged perfusions or perfusions of grafts donated after circulatory death, as it was demonstrated for UW-MPS [3,16]. Nevertheless, the consistency and homogeneity of our findings support the validity of the results for the described collective.

In conclusion, this retrospective propensity score-matched analysis did not reveal any statistically significant differences in postoperative outcomes between donor livers perfused with UW-MPS or HTK during HOPE, thereby rendering them both comparable. However, the HTK group showed an intriguing tendency towards a lower incidence of biliary complications, though this finding remains exploratory due to the study’s retrospective nature and small sample size. Of note, the present investigation is the first comparison of UW-MPS and HTK as perfusion solutions during HOPE. Accordingly, generation of prospective and large-volume data is warranted to further elaborate on a single-solution strategy with reduced cost.

## Figures and Tables

**Table 1:** Cohort comparison.

Parameter	UW N = 23	HTK N = 23	*p* *
	Median (IQR)	Median (IQR)	
Recipient age [years]	60.0 (55.5–63.5)	58.0 (52.5–64.0)	0.50
Recipient sex Male	17	15	0.75
Recipient sex Female	6	8	
Na-MELD [score]	17.0 (13.5–19)	16.0 (14–20)	0.85
Donor age [years]	58.0 (52.0–68.0)	57.0 (45.5–69.5)	0.62
Donor BMI [kg/m^2^]	26.0 (23.0–28.0)	25.0 (23.3–26.0)	0.32
CIT [min]	301.0 (237.5–331.5)	318.0 (284.5–353.0)	0.21
Perfusion time [min]	209.0 (127.5–285.0)	182.0 (155.0–237.5)	0.75
Total preservation [min]	508.0 (440.0–609.5)	476.0 (417.5–556.5)	0.19

* Two-sided Wilcoxon rank sum test (continuous variables) or Fishers exact test (binary variables). According to Shapiro–Wilk normality test and qq-plots variables were not normal distributed. IQR = interquartile range Q1–Q3; Na-MELD = model of end-stage liver disease including sodium levels; BMI = body mass index; CIT = cold ischemia time; min = minutes.

## Data Availability

Data will be made available by the corresponding author upon reasonable request.

## Supplementary Materials

The following supporting information can be downloaded at: https://www.mdpi.com/article/10.3390/jcm15010112/s1, Figure S1: Different parameters used for propensity matching indicate similar distribution for selected patient from the matched patients (Cohort 1, N = 23) compared to the control (Cohort 0, N = 23); Figure S2: Love plot illustrating the absolute standardized mean differences for parameters used for propensity score matching; Figure S3: Minimal detectable-effect table displaying minimal-detectable differences across a range of baseline risks; Table S1: Year of inclusion, perfusate and target arterial pressure.

## Abbreviations

The following abbreviations are used in this manuscript:

95%CI	95% confidence interval
AST	aspartate aminotransferase
ALT	alanine aminotransferase
AP	alkaline phosphatase
BMI	body mass index
CCI	comprehensive complication index
CIT	cold ischemia time
DBD	donation after brain death
EAD	early allograft dysfunction
GGT	gamma glutamyl transpeptidase
HTK	histidine-tryptophan-ketoglutarate
HOPE	hypothermic oxygenated machine perfusion
ICU	intensive care unit
MRCP	magnetic resonance cholangiopancreatography
Na-MELD	sodium model for end-stage liver disease
OR	odds ratio
SCS	static cold storage
UW	University of Wisconsin solution
UW–MPS	University of Wisconsin Machine Perfusion Solution
