# Supplementary material for: HTK Is a Viable UW Alternative for Hypothermic Oxygenated Machine Perfusion of Liver Grafts Supporting a Single-Solution Protocol
Source: J Clin Med. 2025 Dec 24;15(1):112. doi: 10.3390/jcm15010112 (PMC12787218; doi:10.3390/jcm15010112)
Supplement: Supplementary file 1 [file jcm-15-00112-s001.zip › jcm-3990775-supplementary.pdf]

Journal of
Clinical Medicine

MDPI

Article

HTK Is a Viable UW Alternative for Hypothermic Oxygenated Machine Perfusion of Liver Grafts Supporting a Single-Solution Protocol

Jule Dingfelder [1,2,†], David Pereyra [1,2,3†], Moriz Riha [1,2], Nikolaus Becker [1], Laurin Rauter [1], Hubert Hackl [4], Julian Flavio Müller [1], Felix Hammer-Purgstall-Bernd [1], Monika Aiad [1], Jakob Eichelter [1,2], Patrick Starlinger [3,5], Gerd R. Silberhumer [1,2], Andreas Salat [1], Gabriela A. Berlakovich [1], Georg Györi [1] and Thomas Soliman [1,*]

[1] Department of General Surgery, Division of Transplantation, Medical University of Vienna, 1090 Vienna, Austria; david.pereyra@meduniwien.ac.at (D.P.); nikolaus.becker@meduniwien.ac.at (N.B.); gabriela.berlakovich@meduniwien.ac.at (G.A.B.)

[2] Department of General Surgery, Division of Visceral Surgery, Medical University of Vienna, 1090 Vienna, Austria

[3] Department of Surgery, Division of Hepatobiliary and Pancreas Surgery, Mayo Clinic, Rochester, MN 55905, USA

[4] Institute of Bioinformatics, Biocenter, Medical University of Innsbruck, 6020 Innsbruck, Austria; hubert.hackl@i-med.ac.at

[5] Centre of Physiology and Pharmacology, Medical University of Vienna, 1090 Vienna, Austria

* Correspondence: thomas.soliman@meduniwien.ac.at; Tel.: +43-1-40400-68960

† These authors contributed equally to this work.

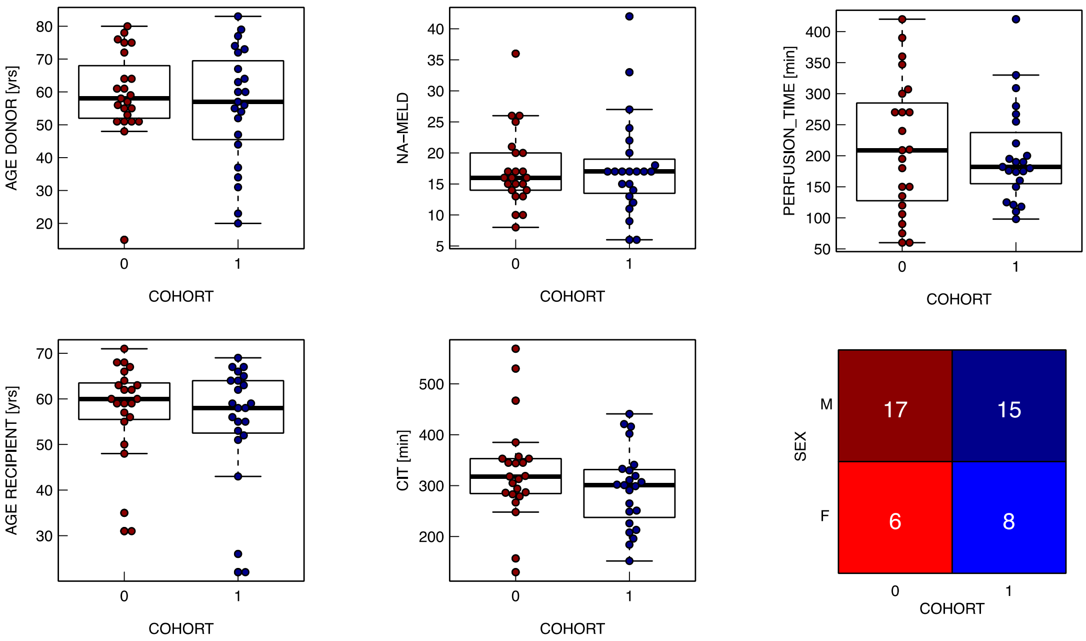

Supplementary Figure S1. Different parameters used for propensity matching indicate similar distribution for selected patient from the matched patients (Cohort 1, N=23) compared to the control (Cohort 0, N=23).

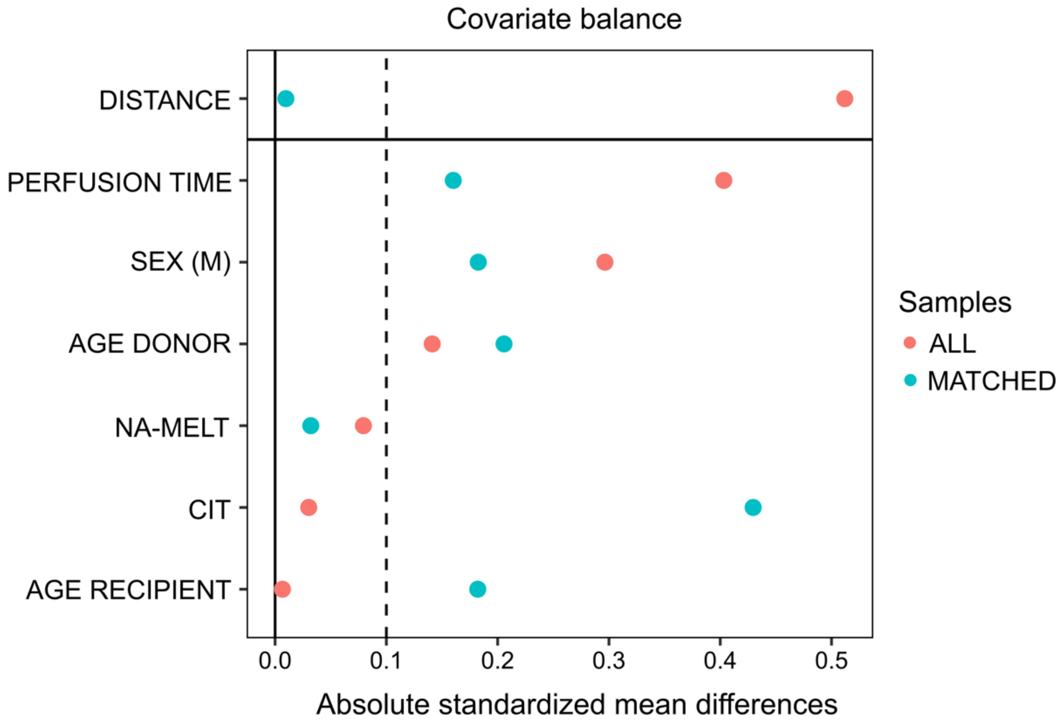

Supplementary Figure S2. Love plot illustrating the absolute standardized mean differences for parameters used for propensity score matching.

baseline_pct	mdd_inc_pp	detectable_inc_pct	mdd_dec_pp	detectable_dec_pct
5	33.1	38.1		
10	36.19	46.19		
20	39.39	59.39		
30	40.26	70.26	29.1	0.9
30.4	40.25	70.65	29.32	1.08
50	37.34	87.34	37.34	12.66

Supplementary Figure S3. Minimal detectable-effect table displaying minimal-detectable differences across a range of baseline risks.

Supplementary Table S1. Year of inclusion, perfusate and target arterial pressure.

Number	Year	Perfusate	Arterial Target Pressure [mmHg]
1	2019	UW	30
2	2019	UW	30
3	2019	UW	no arterial perfusion
4	2019	UW	30
5	2019	UW	30
6	2019	UW	30
7	2019	UW	no arterial perfusion
8	2019	UW	no arterial perfusion
9	2020	UW	30
10	2020	UW	30
11	2020	UW	30
12	2020	UW	30
13	2020	UW	30
14	2020	UW	no arterial perfusion
15	2020	UW	no arterial perfusion
16	2021	UW	no arterial perfusion
17	2021	UW	no arterial perfusion
18	2021	UW	no arterial perfusion
19	2021	UW	no arterial perfusion
20	2022	UW	30
21	2022	UW	30
22	2022	UW	25
23	2022	UW	25
1	2023	HTK	25
2	2023	HTK	25
3	2023	HTK	25
4	2023	HTK	25
5	2023	HTK	no arterial perfusion

6	2023	HTK	25
7	2023	HTK	no arterial perfusion
8	2023	HTK	no arterial perfusion
9	2023	HTK	25
10	2023	HTK	no arterial perfusion
11	2023	HTK	no arterial perfusion
12	2023	HTK	25
13	2023	HTK	no arterial perfusion
14	2023	HTK	25
15	2024	HTK	25
16	2024	HTK	25
17	2024	HTK	no arterial perfusion
18	2024	HTK	25
19	2024	HTK	25
20	2024	HTK	25
21	2024	HTK	25
22	2024	HTK	25
23	2024	HTK	no arterial perfusion
